# Urban-rural disparities in treatment outcomes among recurrent TB cases in Southern Province, Zambia

**DOI:** 10.1186/s12879-019-4709-5

**Published:** 2019-12-30

**Authors:** Simon Mutembo, Jane N. Mutanga, Kebby Musokotwane, Cuthbert Kanene, Kevin Dobbin, Xiaobai Yao, Changwei Li, Vincent C. Marconi, Christopher C. Whalen

**Affiliations:** 1 Department of Epidemiology and Biostatistics, College of Public Health, University of Georgia, Athens, USA; 2 Ministry of Health, Provincial Health Office, Hospital Road, Livingstone, Zambia; 3 Centers for Disease Control and Prevention, Zambia Country Office, Livingstone, Zambia; 4 Division of Infectious Diseases, School of Medicine, Emory University, Atlanta, USA

**Keywords:** Recurrent tuberculosis, Death, Treatment outcomes, Rural, Urban

## Abstract

**Background:**

At least 13–20% of all Tuberculosis (TB) cases are recurrent TB. Recurrent TB has critical public health importance because recurrent TB patients have high risk of Multi-Drug Resistant TB (MDR-TB). It is critical to understand variations in the prevalence and treatment outcomes of recurrent TB between different geographical settings.

The objective of our study was to estimate the prevalence of recurrent TB among TB cases and compare risk of unfavorable treatment outcomes between rural and urban settings.

**Methods:**

In a retrospective cohort study conducted in southern province of Zambia, we used mixed effects logistic regression to asses associations between explanatory and outcome variables. Primary outcome was all-cause mortality and exposure was setting (rural/urban). Data was abstracted from the facility TB registers.

**Results:**

Overall 3566 recurrent TB cases were diagnosed among 25,533 TB patients. The prevalence of recurrent TB was 15.3% (95% CI: 14.8 15.9) in urban and 11.3% (95% CI: 10.7 12.0) in rural areas. Death occurred in 197 (5.5%), 103 (2.9%) were lost to follow-up, and 113 (3.2%) failed treatment. Rural settings had 70% higher risk of death (adjusted OR: 1.7; 95% CI: 1.2 2.7). Risk of lost to follow-up was twice higher in rural than urban (adjusted OR: 2.0 95% CI: 1.3 3.0). Compared to HIV-uninfected, HIV-infected individuals on Antiretroviral Treatment (ART) were 70% more likely to die (adjusted OR: 1.7; 95% CI: 1.2 3.1).

**Conclusion:**

Recurrent TB prevalence was generally high in both urban and rural settings. The risk of mortality and lost to follow-up was higher among rural patients. We recommend a well-organized Directly Observed Therapy strategy adapted to setting where heightened TB control activities are focused on areas with poor treatment outcomes.

## Background

Tuberculosis (TB) remains a major problem in the world and a leading killer among infectious diseases [1]. In 2017 there were 10 million cases of TB and 1.6 million deaths among TB cases [1]. Multi-Drug Resistant TB (MDR-TB) poses a big threat to TB control because of the expensive and limited treatment options and high mortality. MDR-TB is common among patients with recurrent TB, that is individuals who were previously treated with TB drugs for more than one month and once again have been diagnosed with TB disease [2].

It is estimated that 13% of all the TB cases reported to the World Health Organization (WHO) in 2017 were due to recurrent TB [1]. In high Human Immunodeficiency Virus (HIV) burden settings, TB recurrence rates are as high as 20% following standard TB treatment [3, 4]. Recurrent TB can result from reactivation of the original *Mycobacterium tuberculosis* or from re-infection with a different strain [5].

Zambia is a country with a high prevalence of TB (455 cases per 100,000 population) and high prevalence of HIV infection (12% among adults between 15 and 59 years) [6, 7]. The extent of recurrent TB and treatment outcomes of recurrent TB cases have not been fully described. It is important to make distinctions between new and recurrent cases as well as the subgroups of recurrent cases because they are essential for monitoring the TB epidemic and TB program performance [8]. Knowing the prevalence, incidence and treatment outcomes of recurrent TB is important because recurrent TB patients have a high risk of multidrug resistant TB which requires prolonged treatment with more toxic drugs and is associated with higher rates of mortality [9]. To help TB program managers and other scientists involved in TB control to focus strategic activities for recurrent TB detection, treatment and consequently prevent MDR-TB, the scientific world must understand the variations in recurrent TB treatment outcomes between different subpopulations and geographical settings.

The burden of TB disease is generally considered to be higher in urban settings than rural settings due to overcrowding, high HIV prevalence and occupational transmission [6]. However it is not known whether a difference exists between the prevalence of recurrent TB in rural and urban settings. It is critical to understand the difference in the prevalence of recurrent TB between rural and urban settings because of the differences in the social and economic standards as well as differences in the accessibility and quality of health care between the 2 settings. Health care service delivery is better in urban populations because health facilities are easy to reach whereas rural patients must travel long distances [10, 11]. Additionally, urban settings have more skilled health care providers with more clinical experience in the management of TB and HIV and access to better diagnostics. In contrast, most TB patients in urban settings are from deprived communities and social disadvantages such as slums which may contribute to poorer TB treatment outcomes [10].

We examined the prevalence of recurrent TB among TB cases notified to the National TB Program (NTP) in southern province of Zambia. We assessed for risk factors associated with death, loss to follow up and failure among recurrent TB patients accessing treatment at health facilities in rural and urban settings.

## Methods

### Study design

A retrospective cohort study was conducted by analyzing data collected in the NTP in southern province of Zambia.

### Study setting and population

The study was conducted in a predominantly rural setting (85%) with a high TB case notification rate (> 300 cases per 100,000 population per year) [12].

All adults and children who were treated for recurrent TB between January 2006 and June 2014 were included in the analysis. Recurrent TB was defined as a case of TB disease diagnosed in a patient who had been treated before for more than 1 month with TB drugs [2].

### Study exposure

The study assessed factors associated with death, treatment failure and loss to follow up (LTFU) among patients treated for recurrent TB. The main exposure was health facility location classified as either rural or urban. The definition of rural or urban was based on the Ministry of health and Central Statistical Office of Zambia classification of health facilities [11]. A rural area is a location where the source of livelihood for at least 75% of the population is agriculture and agriculture allied activities and the health facility in the area serve a catchment population of less than 30,000 [11]. Urban health facilities are facilities that are located in urban settings and serve catchment populations of over 30,000 [11].

### Study outcomes

The primary outcome was all-cause mortality which is death from TB disease or any other cause before the patient successfully completed treatment. Death was verified by the attending physician through death certificate or verbal report from treatment supporters or the patient’s next of kin.

The secondary outcomes were LTFU and treatment failure. LTFU was defined as a patient having missed more than two months of consecutive doses [2]. Treatment failure was defined as patient being smear positive after more than 5 months of treatment [2].

### Covariates

The following clinical and demographic characteristics were evaluated as risk factors for death: age, sex, HIV status and ART in TB/HIV co-infected patients, treatment delay and clinical classification of disease. Clinical Pulmonary TB was a situation where the attending clinician made a diagnosis of pulmonary TB and decided to give the patient a full course of anti-TB treatment without bacteriological confirmation; Bacteriologically confirmed TB was when the diagnosis and treatment was supported by biological specimen such as by Acid-Alcohol Fast Bacilli (AAFB) smear microscopy, culture or GeneXpert [2].

### Data collection and data integrity

We abstracted clinical and demographic data on the TB cases that had been recorded in the NTP register at every outpatient visit. Data entries were subjected to quality control, including removal of duplicate data entries, outliers in continuous variables, date parameters and ambiguous or erroneous entries in categorical entries. Verification of extracted records with source data was done in a randomly selected subset of 10 patients for every 100 entries from each site. After quality control, all available records for TB patients registered at the participating health centers between January 2006 and January 2013 were screened for inclusion.

### Statistical analysis

Data were described using frequency counts and percentages for categorical variables, means and standard deviations for normally distributed continuous variables. In the primary analysis we estimated the odds ratio and the 95% confidence intervals for death among rural cases compared with urban cases. We first estimated the crude odds ratio and then stratified to assess for confounding and interaction. For associations between explanatory variables and the primary and secondary outcomes we used mixed effects logistic regression since participant data was organized at more than one level: district, health facility and rural/urban setting. The data were cleaned and analyzed using Stata v.14.

### Ethical considerations

Ethical approval was obtained from the Macha Research Trust ethics committee, the University of Georgia institutional review board and regulatory authority from the Zambia National Health Research Authority. Before commencing data abstraction administrative permission was obtained from the provincial health office in southern province.

## Results

### Case enrollment

**Fig. 1:**
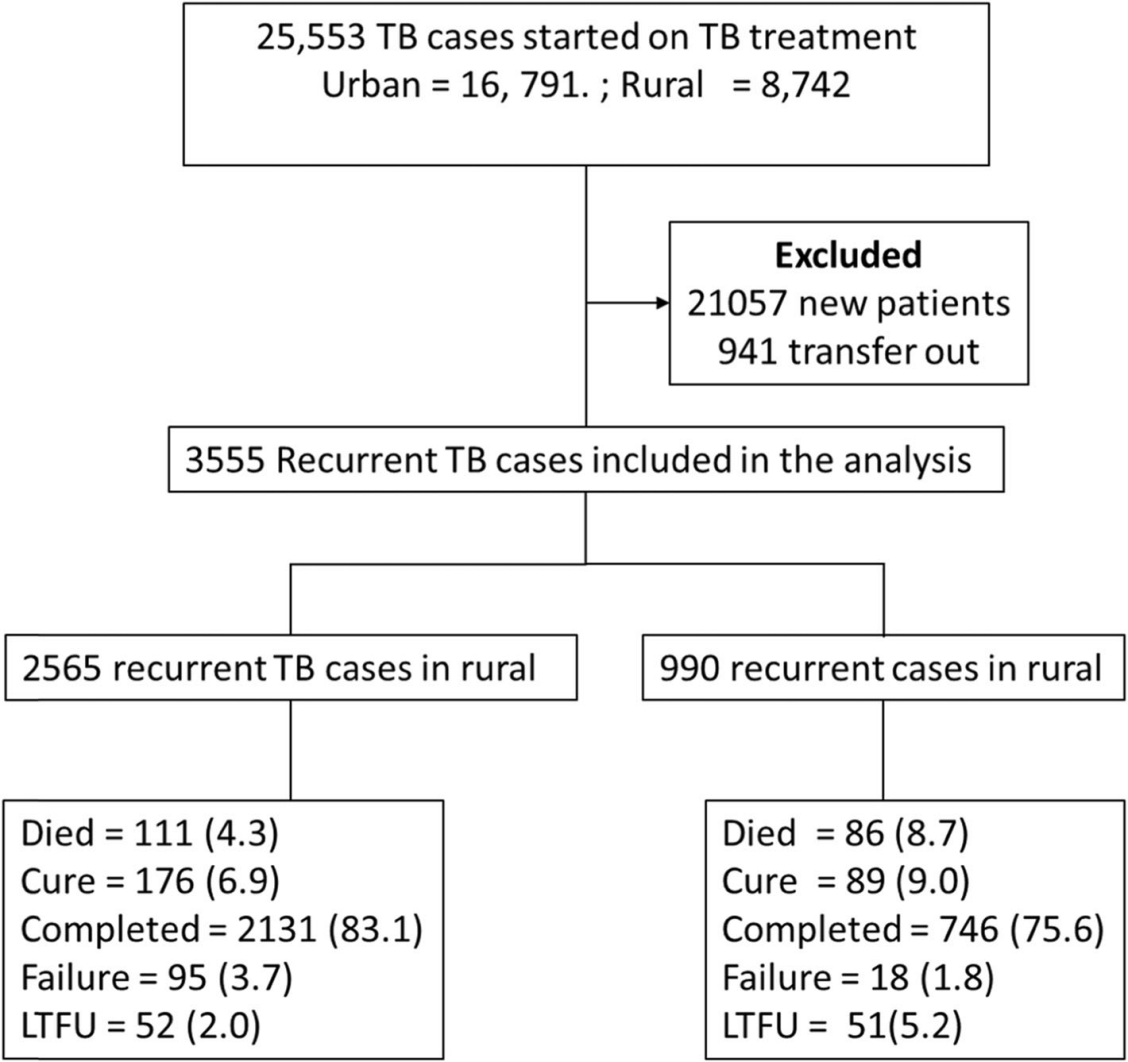
Consort diagram showing the selection of recurrent TB patients and treatment outcomes in rural and urban setting

Data from 25,533 TB patients was abstracted and after excluding new TB cases and patients who transferred out, we analyzed 3555 recurrent TB cases. Of these recurrent TB cases 990 (27.8%) were in rural areas and 2565 (72.2%) were in urban areas (Fig. 1).

### Prevalence of recurrence among TB cases

The overall prevalence of recurrent TB before excluding cases that transferred out was 14% (95% CI; 13.5 14.4). In urban setting the prevalence was 15.3% (95% CI: 14.8 15.9) and 11.3% (95% CI: 10.7 12.0) in rural areas (Table 1). TB recurrence in HIV negative TB cases was 10.0% (*n* = 499; 95% CI: 9.2 10.9) and 16.1% (*n* = 2216; 95% CI: 15.5 16.7) in HIV seropositive positive recurrent TB cases. In men prevalence of recurrent TB was 13% (*n* = 1506; 95% CI: 12.4 13.6) and 14.9% (*n* = 2049; 95% CI: 14.3 15.5) in women.

**Table 1:** Prevalence of Tuberculosis retreatment cases by different characteristics among patients who were treated for tuberculosis

Patient characteristics	total number of TB cases	recurrent cases	Prevalence in % (95% CI)
Overall	25,533	3566	14.0 (13.5 14.4)
Urban site	16,791	2574	15.3 (14.8 15.9)
Rural site	8742	992	11.3 (10.7 12.0)
Age			
10 & below	3259	280	8.6 (7.7 9.6)
11–18	2641	272	10.3 (9.1 11.5)
19–55	16,835	2614	15.5 (15 16.1)
56 &older	2769	400	14.5 (13.1 15.8)
Sex			
Female	11,569	1506	13 (12.4 13.6)
Male	13,726	2049	14.9 (14.3 15.5)
HIV status			
negative	11,709	1382	11.8 (9.2 10.9)
positive	13,795	2216	16.1 (15.5 16.7)

Note: In this table we estimate the prevalence using the overall number of cases before excluding cases who did not have the outcomes of interest at the end of follow (patients who transferred out and the TB treatment outcome was not documented

### Demographic and clinical characteristics

The mean was 36.3 years (Standard deviation (SD) = 14.8) and most of the recurrent TB cases were male (*n* = 2019; 56.9%) Of the total cases 2745(77%) were recurrent cases due to relapse, 31 (0.9%) were recurrence after LTFU, 29 (0.8%) were recurrence after failure and 106 (3.0%) were classified as others (Table 2).

**Table 2:** Clinical and demographic characteristics of recurrent TB cases treated for drug susceptible TB in Zambia, southern province

Characteristic	Level	Total	Urban areas	Rural areas
		*n* (percent)	*n* (percent)	*n* (percent)
Sex	Female	1482 (41.7)	1045 (40.7)	437 (44.1)
	male	2019 (56.9)	1473 (57.4)	546 (55.2)
	Missing	54 (1.52)	47 (1.8)	7 (0.7)
Age group^a^	10 & below	128 (3.6)	93 (3.6)	35 (3.5)
	11–18	140 (3.9)	103 (4.0)	37 (3.7)
	19–55	2185 (79.2)	2064 (80.5)	751 (75.9)
	56 & older	403 (11.3)	259 (10.1)	144 (14.6)
	Missing	69 (1.9)	46 (1.8)	23 (2.3)
Microbiological confirmation	clinical	2519 (70.9)	1874 (73.1)	639 (64.6)
	confirmed	1036 (29.1)	689 (26.9)	347 (35.1)
Reason for retreatment	Relapse patients	2745 (77.2)	2126 (82.9)	619 (62.5)
	Other	644 (18.1)	330 (12.9)	314 (31.7)
	Treatment after LTFU patients	31 (0.8)	28 (1.1)	3 (0.3)
	Treatment after failure patients	29 (0.82)	15 (0.6)	14 (1.4)
	Missing	106 (3.0)	66 (2.6)	40 (4.0)
Site of disease	Pulmonary	2937 (82.6)	2086 (81.3)	851 (86.0)
	Extra-pulmonary	537 (15.1)	418 (16.3)	119 (12.0)
	Missing	81 (2.3)	61 (2.4)	20 (2.0)
Treatment Delay	14 days & less	3135 (88.2)	2286 (89.1)	849 (85.8)
	15 days and more	420 (11.8)	279 (10.9)	141 (14.2)
Calendar year	< 2010	1884 (53)	1336 (52.1)	548 (55.4)
	2010 & after	1671 (47)	1229 (47.9)	442 (44.7)
Facility type	Hospital	1353 (38.1)	926 (36.1)	427 (43.1)
	Health Center or clinic	2202 (61.9)	1639 (63.9)	563 (56.9)
HIV status	Negative	1382 (38.9)	853 (61.7)	529 (38.3)
	Positive	2173 (61.1)	1712 (78.8)	461 (21.2)

^a^Age group (< 10, 11–18, 19–55 and > 55) classification was based a WHO pediatric treatment guidelines and other previous studies of TB treatment outcomes [13, 14].

Of all the HIV seropositive cases of recurrent TB that were analyzed 1712 (78.8%) cases were in urban areas whereas 461 (21.2%) were in rural areas (Table 2). Most patients received treatment within the first 14 days of registering in the TB clinic (*n* = 3135; 88.2%).

### Treatment outcomes

Of the 3555 patients who were included in the analysis 265 (7.5%) were cured and 2877 (80.9%) completed treatment (Table 3). In the urban areas 111 (4.3%) cases died, 95 (3.7%) cases experienced treatment failure and 52 (2.0%) were LTFU. In the rural areas 51 (5.2%) died, 18 (1.8%) experienced TB treatment failure and 51 (5.2%) were LTFU.

**Table 3:** TB treatment outcomes among recurrent TB cases in rural and urban settings in southern province, Zambia

Treatment outcomes	Total number (percent)	Urban	Rural
		n (percent)	n (percent)
Cure	265 (7.5)	176 (6.9)	89 (9.0)
Completed	2877 (80.9)	2131 (83.1)	746 (75.4)
Failure	113 (5.5)	95 (3.7)	18 (1.8)
Died	197 (2.9)	111 (4.3)	86 (8.7)
LTFU	103 (3.2)	52 (2.0)	51 (5.2)
Total	3555	2565	990

### Univariable and multivariable analysis results

In both the univariable and multivariable analysis place of TB treatment was statistically associated with all-cause mortality (Table 4). After adjusting for sex, age group, microbiological confirmation, and HIV status, recurrent TB cases treated in the rural settings were 70% more likely to die while on TB treatment. (aOR: 1.7, 95% CI: 1.2 2.7). HIV positive patients on ART were 70% more likely to die when compared to HIV negative patients (aOR 1.7; 95% CI: 1.4–3.7).

**Table 4:** Estimates of the risk of death (OR) in recurrent TB patients in southern province Zambia according to demographic and clinical characteristics

Characteristics	Total	Died	Crude	Adjusted
			OR (95% CI)	OR (95% CI)
Location				
Urban	2574	111	1	1
Rural	991	86	2.1 (1.5 2.8)	1.7 (1.2 2.7)
Sex				
Female	1482	112	1	1
Male	2019	85	0.5 (0.4 0.7)	0.6 (0.4 1.0)
Age group				
Below 55	2721	171	1	
> 55	403	26	1.0 (0.8 1.3)	
Microbiological confirmation				
clinical	2519	62	1	
confirmed	1036	135	1.1 (0.8 1.5)	0.6 (0.4 1.0)
Site of disease				
pulmonary	2937	169	1	
Extra-pulmonary	537	24	0.8 (0.5 1.2)	
Treatment Delay				
< =14 days	3135	174	1	
> 15 days	420	23	1.2 (0.6 1.5)	
Facility type				
Calendar				
< =2010	1884	106	1	
> 2010	1671	91	0.9 (0.7 1.3)	
HIV				
Seronegative	1382	367	1	
Seropositive + ART	1187	610	1.6 (1.4 3.7)	1.7 (1.4 3.7)
Seropositive no ART	986	114	2.3 (1.8 6.8)	2.3 (1.6 6.0)

For secondary outcomes of LTFU and TB treatment failure analyzed separately rural areas had worse TB treatment outcomes. After adjusting for calendar year of treatment, HIV and ART status recurrent TB patients in the rural areas where twice more likely to be LTFU as compare to those in urban areas (aOR 2.0, 95% CI: 1.3 3.0) (Table 5). For recurrent TB treatment failure, patients in rural areas were 70% more likely to experience treatment failure (aOR 1.7, 95% CI: 0.7 5.1) (Table 6).

**Table 5:** Estimates of the risk of LTFU (OR) in recurrent TB patients in southern province Zambia according to demographic and clinical characteristics

Characteristic	total	LTFU	Crude OR (95% CI)	Adjusted OR (95% CI)
Setting				
Urban	2565	52	1.0	
Rural	990	51	1.9 (1.1 3.4)	2.0 (1.3 3.0)
Sex				
Male	2019	53	1.0	
Female	1482	50	0.8 (0.5 1.2)	
Age group				
Below 55	2721	90	1.0	
Above 55	403	13	0.9 (0.8 12)	
Calendar year				
Before 2010	1884	53	1.0	
After 2010	1671	50	0.99 (0.7 1.5)	1.1 (0.7 1.5)
Treatment delay				
Less than 14 days	3135	92	1.0	
14 days and more	420	11	0.8 (0.4 1.4)	
Classification of TB				
Microbiology confirmed	1036	40	1.0	
clinical	2460	63		
HIV and ART status				
HIV negative	1382	57	1.0	
HIV seropositive on ART	1187	39	0.9 (0.6 1.3)	0.8 (0.5 1.2)
HIV seropositive no ART	986	7	0.9 (0.7 1.4)	0.9 (0.8 1.4)

**Table 6:** Estimates of the risk of treatment failure (OR) in recurrent TB patients in southern province Zambia according to demographic and clinical characteristics

Characteristic	total	Treatment failure	Crude OR (95% CI)	Adjusted OR (95% CI)
Setting				
Urban	2565	95	1.0	
Rural	990	18	1.4 (0.5 3.9)	1.7 (0.7 5.1)
Sex				
Male	2019	60		
Female	1482	50	0.8 (0.5 1.2)	
Age group				
Below 18 years	268	10	1.0	
18–55	2815	86	0.7 (0.4 1.5)	
Above 55	403	17	1.3 (0.5 2.9)	
Calendar year				
Before 2010	1884	54	1.0	
After 2010	1671	59	1.2 (0.8 1.8)	
Treatment delay				
Less than 14 days	3135	90		
14 days and more	420	23	2.1 (1.2 3.4)	1.7 (0.9 2.4)
Classification of TB				
Microbiology confirmed	1036	63		
clinical	2460	50	0.3 (0.2 0.4)	0.4 (0.2 0.6)
HIV and ART status				
HIV negative	1382	45	1.0	
HIV seropositive on ART	1187	27	0.7 (0.5 1.4)	0.7 (0.4 1.2)
HIV seropositive no ART	986	41	0.8 (0.4 1.2)	1.0 (0.6 1.7)

## Discussion

In this retrospective cohort of TB patients, the overall prevalence of recurrent TB of 14% is high and similar to other sub-Saharan Africa countries [15]. It is higher in urban (15.3%) than in rural areas (11.3%). Recurrent TB prevalence was higher among HIV seropositive individuals than HIV seronegative individuals (16.1 vs 11.8). Recurrent TB cases receiving treatment in rural areas were 70% more likely to die when compared to patients treated in urban areas. The risk LTFU was twice higher in rural areas than urban areas.

Overall the prevalence of recurrent TB was high and similar to other sub-Saharan African settings with high HIV prevalence [3]. The prevalence was slightly higher in urban areas than rural areas. High prevalence of recurrent TB can be driven by either a high rate of exogenous re-infection or high rate of relapse of the initial *M. tuberculosis* infection. In this study, high prevalence of TB in the general population and among HIV seropositive individuals provides a suitable environment for high exposure to *M. tuberculosis* and consequently a high potential of re-infection. Because of the limited laboratory capacity, distinction between exogenous re-infection and true relapse cases could not be made. However, previous molecular studies in similar settings have demonstrated that a large proportion of recurrent TB cases among HIV patients are caused by exogenous reinfection. In a cohort of South African gold-mine workers, HIV-1 infection was strongly associated with re-infection but not with relapse. This finding has programmatic and public health consequences because even with effective TB treatment regimens TB recurrence can be more common in population with high HIV prevalence as long as exposure to *M. tuberculosis* is high [3, 16].. Another study in Uganda showed that recurrent TB occurring more than 2 years after completing TB treatment was mainly due to re-infection whereas recurrence occurring within 2 years of treatment was due to relapse. Unfortunately, the NTP registers do not capture the dates when the last TB treatment was completed and hence it was not possible to ascertain the time interval between TB episodes [15].

The risk of death was higher in rural areas than urban areas. Generally, patients in rural areas have worse TB treatment outcomes than patients in the urban areas including LTFU [10, 17]. Our study presents data on recurrent TB and provides a valid assessment of recurrent TB treatment outcomes presented separately for death, treatment failure and LTFU. For recurrent TB patients understating the magnitude of LTFU is critical because recurrent TB patients who are LTFU are likely to progress to MDR-TB and are a potential risk to further transmission of the multi-drug resistant *M. tuberculosis*. Although the prevalence of recurrent TB was lower in rural than urban settings, LTFU was higher in rural settings. This implies that rural settings are at increased risk of creating next generation of MDR-TB cases.

Most studies that have assessed risk factors associated with unfavorable TB treatment outcomes found that urban areas generally have better outcomes because of better organized patient follow up systems and relatively easy access to health care services [18–20]. A qualitative study conducted in Uganda highlighted geographical barriers in rural areas as one of the barriers for delivery of routine TB diagnostic and treatment services [19]. Interviews highlighted physical remoteness of their homes from the clinic and the tough terrain encountered during travel as the principal barrier to accessing timely TB evaluation and treatment [19]. Challenges in accessing diagnostic and treatment services have potential of influencing TB treatment outcomes and increasing mortality among TB patients. Additionally, urban settings have a high prevalence of TB and HIV and have more healthcare providers as compared to rural settings [11]. Therefore health workers in the urban areas have more clinical experience in managing TB and HIV.

The strength of this study is that it describes recurrent TB cases from a large cohort of TB cases at multiple rural and urban sites in a setting with high prevalence of both TB and HIV. Additionally, the paper highlight some of the gaps in the program and clinical case management of recurrent TB. The gaps herein highlighted are likely to be found in most NTPs in sub-Saharan Africa.

### Limitations

As in any real-world data or observational studies our analysis was subject to some limitations. An obvious weakness of this analysis was errors in the recorded data and missing which could not be validated. To address this potential selection bias, we conducted a sensitivity analysis by comparing baseline characteristics of those included in the analysis and those excluded. The two sample populations were similar on most aspects. Hence selection bias was very unlikely. The data used in this analysis program data meant for clinical care and follow up of patients. It does not capture a lot of social and demographic factors. Therefore, risk factors associated with the prevalence of recurrent TB could not be determined.

### Public health relevance

We have shown that recurrent rates of TB are high in both rural and urban settings with rural areas having worse treatment outcomes as compared to urban areas. Therefore, a well-organized patient monitoring system such as effectively administered Directly Observed Treatment Short course (DOTS) can help early detection of recurrent cases and reduce mortality and LTFU. DOTS must be adapted to the setting.

## Conclusion

Prevalence of recurrent TB was high in both rural and urban areas in the southern part of Zambia. The prevalence was higher among TB patients who access treatment at health facilities in urban areas than rural areas. However, TB recurrent cases accessing treatment in rural areas experienced higher mortality and worse treatment outcomes as compare to those accessing treatment in urban areas. We strongly recommend effective DOTS adapted to the setting where heightened DOTS activities are focused in areas or communities with poor TB treatment outcomes.

## Data Availability

The data that support the findings of this study are available from the national TB control program at the provincial health office in southern province Zambia, but restrictions apply to the availability of these data, which were used under license for the current study and are currently not publicly available. Data can be made available for other authors upon request and with the permission and approval of the Ministry of Health and the Zambia National Research Authority.

## Abbreviations

AAFB: Acid-alcohol fast bacilli
aOR: Adjusted odds ratio
ART: Anti-retroviral therapy
CI: Confidence interval
DOTS: Directly observed treatment short course
HIV: Human immunodeficiency virus
LTFU: Lost to follow up
MDR: Multi drug resistant
NTP: National tuberculosis control program
OR: Odds ratio
SD: Standard deviation
WHO: World Health Organization
